# Case of Procidentia Uteri during Labour, in Which Artificial Means Were Necessary to Effect Delivery, with Subsequent Replacement of the Uterus, and Complete Recovery

**Published:** 1845-06

**Authors:** John M. B. Harden

**Affiliations:** Liberty County, Georgia


*Case of Procidentia Uteri during Labour, in which artificial means were necessary to effect Delivery, with subsequent replacement of the Uterus, and complete Recovery.* By JOHN M. B. HARDEN, M. D., of Liberty County, Georgia.—The following case, the narration of which I have received from my friend, Dr. Raymond Harris, of Bryan county, is so very curious and interesting, that I have considered it worthy of permanent record, and therefore send it, with the request that it be inserted in the next number of the Southern Medical and Surgical Journal. Although not occurring under my own observation, yet, from the known character of the gentleman who has furnished it to me, I have no hesitation in vouching for the general accuracy of the details.

In April, 1829, a negro woman, belonging to Capt. George Rentz, of McIntosh county, was taken in labour. She was about 40 years of age, of good constitution, mother of several children, and so far as is known, not subject to any previous prolapsus, or other disease of the womb. Something unusual and anomalous having occurred during the progress of the labour, Dr. Harris was sent for. He found her, on his arrival, in the following condition. She was lying on her back, with the whole gravid uterus between her thighs, retained only by the ligaments, which were much stretched, but not ruptured, and discharging from its external surface a serous or sanious fluid. The woman had been in this condition for about 24 hours. She had had no pain since the descent of the uterus, and was complaining of none at this time. The liquor amnii had been discharged. After a careful examination, no motion or other sign of life in the fœtus could be perceived. The uterus appeared to be in a perfectly quiescent state, without any disposition to contract. The os tincæ was barely dilated sufficiently to allow the introduction of *two fingers.* Finding it absolutely necessary to relieve her as soon as possible, the Doctor proceeded to deliver her by artificial means. He opened the head of the child with a suitable instrument, and then, having an assistant to hold and support the uterus, he introduced his hand, and by careful *traction*, succeeded in removing its contents. There was very little pain during his manipulations. He now returned the womb, which had scarcely contracted at all, and advising the recumbent position, left her. She had a *very good* "*getting up*," and two years ago, the Doctor learned, *was in good health.*

Remarks.—Cases of the above character must be of very rare occurrence. I have not been able to lay my hands on more than two bearing any resemblance to it. One is noticed in West's Report, published in the British and Foreign Medical Review, for April, 1844,* and occurred in the practice of Dr. Perfetti. In this case, however, the procidentia was not complete, the uterus only reaching "something more than six fingers breadth beyond the external parts." The woman had been subject to prolapsus "ever since she was fifteen years old." The other had been communicated to the Dublin Medical Press, by Dr. Darbey, of Drogheda.† The woman was 42 years of age, and had had prolapsus uteri for some years. This was her seventh pregnancy. "On examination, Dr. D. found the uterus *lying between the patient's thighs*, presenting a livid appearance, and the os uteri having a dry feel, and no symptoms of dilatation. The labour pains *were strong*, *with violent cramps in the lower extremities*."

2. The treatment of these cases seems to have been governed by the circumstances attending them. In our first case, the os uteri was "*so hard and undilatable*," that Dr. Perfetti deemed it necessary to *make incisions into it.* He then *introduced the forceps and extracted the child.* The mother recovered. Dr. Darbey "took thirty ounces of blood from the arm, and administered the following draught: ℞. Aq. Menth. Pip. ℥iss.; Tr. Opii. Acet. gtt. 4; Syrup Cort. Aurant. ʒij. M.—which procured rest, and checked the cramps and other bad symptoms. After a comfortable repose of two hours, *labor*

* Am. Jour. Med. Sciences, N. S. vol. 8, p. 257.

† Am. Jour. Med. Sciences, N. S. vol. 9, p. 232.

*pains returned*, the os uteri gradually and steadily dilated, and a healthy, *but small sized child, was born.* The placenta followed after a short time, and the uterus *being replaced and suitably secured, nothing untoward followed.*" In our case the dilatation was effected by the hand, after having lessened the dimensions of the head; and certainly this method should *always be preferred to incisions, unless it be found impracticable.*

3. There is an important physiological fact to be gleaned from these cases: namely, the *power of the abdominal muscles in effecting delivery;* and the case which we have now related shows plainly *that parturition may be carried through by the action of these muscles alone, without the concurrence of uterine contraction*, and naturally suggests the question, which plays the most important part in Labour? Any one who has ever had his hand in the uterus during a labour pain, must know that there is most powerful action of muscles *somewhere*, and he would no doubt be inclined to refer it to the uterus itself—but may not the most of this force arise from the abdominal muscles acting through the parieties of the *uterus?* and may not the mechanism of labour, in this regard, be similar to the mechanism of vomiting? For our part, we are very much inclined to adopt the affirmative; while at the same time we admit that the uterus has *an independent action and power of its own*, and that in every healthy labour, this action and contraction march *pari passu* with the expulsion of its contents.—*Southern Med. and Surg. Journal.*

